# *Sarcocystis cristata* sp. nov. (Apicomplexa, Sarcocystidae) in the imported great blue turaco *Corythaeola cristata* (Aves, Musophagidae)

**DOI:** 10.1186/s13071-020-04553-w

**Published:** 2021-01-18

**Authors:** Ondřej Máca, David González-Solís

**Affiliations:** 1 Department of Pathology and Parasitology, State Veterinary Institute Prague, Sídlištní 136/24, 165 03 Prague 6, Czech Republic; 2 Department of Zoology and Fisheries, Faculty of Agrobiology, Food and Natural Resources, Czech University of Life Sciences Prague, Kamýcká 129, 165 00 Prague Suchdol, Czech Republic; 3 El Colegio de la Frontera Sur, Chetumal. Av. Centenario km 5.5, 77014 Chetumal, Quintana Roo Mexico

**Keywords:** Africa, *Sarcocystis*, Aves, Molecular characterization, New species

## Abstract

**Background:**

Species of *Sarcocystis* are parasitic protozoa in poikilothermic and homeothermic animals. Out of the 26 valid species in birds as intermediate hosts, none has been reported in those of the order Musophagiformes, such as the great blue turaco *Corythaeola cristata* (Vieillot, 1816), which is a bird endemic to Central and Western Africa. The examination of great blue turacos imported from the Central Africa Republic to Czech Republic allowed the morphological and molecular characterization of a new species of *Sarcocystis*.

**Methods:**

Four turacos imported from the Central Africa Republic to a private breeder (Czech Republic) underwent parasitological examination for the presence of sarcocysts through wet mounts of breast, heart and leg muscles. Found parasites were molecularly and histologically studied by four loci (*18S* rRNA, *28S* rRNA, ITS1 and *cox1*) and haematoxylin and eosin staining, respectively.

**Results:**

Three out of four examined birds harboured numerous sarcocysts in the breast and leg muscles. No macroscopic lesions where observed. Sarcocysts were microscopic, elongate and ribbon-shaped with a wall characterised by the presence of finger-shaped villar protrusions and filled with numerous elongate, banana-shaped bradyzoites, 11.87–14.84 × 2.05–2.92 µm in size. The new species was most closely related to *Sarcocystis* *albifronsi*, *Sarcocystis* *anasi*, *Sarcocystis* *atraii*, *Sarcocystis* *chloropusae*, *Sarcocystis* *rileyi*, *Sarcocystis* *wenzeli* and *Sarcocystis* sp. isolate from chicken in the four loci.

**Conclusions:**

To our knowledge, this is the first species of *Sarcocystis* found in a musophagiform bird worldwide. Genetically, *S. cristata* sp. nov. represents a distinct species. Phylogenetic analyses are useful for predicting potential definitive hosts of the new *Sarcocystis* species.
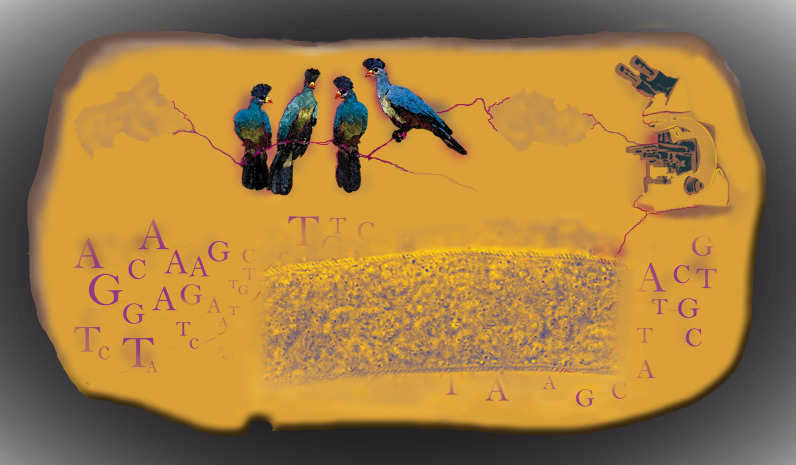

## Background

Species of the genus *Sarcocystis* Lankester, 1882, are parasitic protozoa with an indirect life cycle using poikilothermic and homeothermic animals as definitive and intermediate hosts. The latter includes mammals, birds, marsupials and poikilothermic animals where most sarcocysts are found in the striated muscles of the heart, tongue, oesophagus, diaphragm and skeletal muscles as well as in the smooth muscles of intestine [1]. To date, 16 bird orders (e.g. Anseriformes, Galliformes, Passeriformes, etc.) have been reported worldwide as intermediate hosts of 40 species of *Sarcocystis*, of which 26 are valid, while the rest are invalid or species inquirendae [1, present paper]. However, there still are no records of sarcocysts in birds of the order Musophagiformes, such as the great blue turaco *Corythaeola cristata* (Vieillot, 1816) (Musophagiformes, Musophagidae), which is a bird endemic to Central and Western Africa. It has been scarcely parasitologically studied, with records mostly on haematozoan parasites (genera *Haemoproteus*, *Leucocytozoon*, *Microfilaria* and *Trypanosoma*) [2–8], but nothing on *Sarcocystis*. During the examination of great blue turacos imported from the Central Africa Republic to Czech Republic, sarcocysts of an apparently new species of *Sarcocystis* were found in the breast and legs muscles. The morphological and molecular characterization of those specimens is presented here as well as the role as an intermediate host in the life cycle of a *Sarcocystis* species.

## Methods

In 2019, four frozen turacos in poor body condition were sent to the State Veterinary Institute (SVI) Prague for necropsy. These birds were imported from the Central Africa Republic to a private breeder (Czech Republic) where they died after s few days in quarantine. Later, the birds were examined for parasites under light microscopy (LM) through wet mounts of breast, heart and leg muscles for the presence of sarcocysts. The flotation method was also used to detect the presence of parasites in the digestive tract. Both methods were carried out using a Leica DMLB optical microscope with a Leica DFC420 digital camera (Leica Microsystems, Wetzlar, Germany) equipped with Nomarski differential interference contrast. Found parasites were transferred to Eppendorf tubes for DNA extraction. For histology, muscles with sarcocysts were fixed in 10% formalin solution, and tissue sections were stained with haematoxylin and eosin. All measurements are given in micrometres, unless otherwise mentioned.

Genomic DNA was extracted by glass bead disruption from six isolates of sarcocysts collected from breast and leg muscles using the QIAamp^®^ Fast DNA Stool Mini Kit (Qiagen, Hilden, Germany) according to the manufacturer’s recommendations. DNA was stored at − 20 °C until use in polymerase chain reaction (PCR) assays of *18S* rRNA, *28S* rRNA, ITS1 and *cox1* loci. PCR and nested-PCR were carried out by using primers for *18S* rRNA (A2F/Primer BSarc and Fext/Rext; Fint/Rint) [9, 10], *28S* rRNA (KL_P1R/KL_P1F and KL1/LS2R; LS1F/KL3) [11–13], ITS1 region (ITS-F/ITS-R; SU1F/5.8SR2) [11, 13] and *cox1* genes (SF1/SR10 or SF1/SR5) [14, 15]. Amplification was carried out in a final volume of 25 μl (20 μl reaction mixture and 5 μl DNA extract) comprising 1× Green GoTaq^®^ Flexi Buffer, 2.5 mM of MgCl_2_, 0.625 U of GoTaq^®^ G2 Flexi DNA Polymerase (Promega, Madison, WI, USA), 0.2 mM dNTP mix (Bioline, London, UK) or GoTaq^®^ G2 Colorless Master Mix (Promega, Madison, WI, USA), 0.4 μM of each primer, DNA template and nuclease-free water. PCR amplification of isolated DNA samples plus positive and negative controls was performed with the following cycling conditions: an initial denaturation step at 95 °C for 5 min; 35 cycles of 95 °C for 30 s, 52–60 °C for 30 s and 72 °C for 1 min; a final extension step at 72 °C for 10 min. The PCR products were later analysed by electrophoresis in 1‒1.5% agarose gel and visualised by ethidium bromide staining. The PCR products were purified using the High Pure PCR Product Purification Kit (Roche Diagnostics, Mannheim, Germany) or the ExoSAP-IT™ Express PCR Product Cleanup Reagent Kit (Thermo Fisher Scientific) according to the manufacturer’s protocol. Cleaned amplicons were sequenced through the commercial company Eurofins Genomics (Ebersberg, Germany) using both forward and reverse primers. The nucleotide sequences of the four loci derived in this study have been deposited in the GenBank database under accession numbers MT676453–MT676455 and MT681118.

The newly generated sequences were analysed and edited using FinchTV software (Geospiza Inc., Seattle, WA) and compared with published sequences of the valid species of *Sarcocystis* across the GenBank NCBI database using BLAST (Basic Local Alignment Search Tool). Sequences were aligned using MAFFT software version 7 [16], and phylogenetic trees were constructed using the MEGA X [17]. The neighbour-joining (NJ) method and Tamura three-parameter model were used for *18S* rRNA and *cox1* genes, while the maximum likelihood method (ML) and Hasegawa-Kishino-Yano model were used for the *28S* rRNA gene and ML and Tamura-Nei model for the ITS1 region. For the NJ method, evolutionary distances were computed using the Tamura three-parameter method (T92 + G) from the selected 1716 aligned positions with 19 nucleotide sequences of *18S* rRNA and from the selected 1013 aligned positions with 18 nucleotide sequences of *cox1* gene. For ML, they were performed by applying the Hasegawa-Kishino-Yano model (HKY + G + I) from the selected 1330 aligned positions with 23 nucleotide sequences for the *28s* rRNA gene and Tamura-Nei model (TN93 + G + I) from the selected 1460 aligned positions of 23 nucleotide sequences for the ITS1 region. A bootstrap test of phylogeny was computed based on 1000 replicates for all loci, and NJ and ML trees were rooted with *Toxoplasma gondii*.

## Results

Three out of four examined birds (prevalence: 75%) harboured at least one sarcocyst. No macroscopic lesions were observed in the organs of infected birds. However, sarcocysts were found in breast and leg muscles in similar numbers (three sarcocysts per gram). Heart muscles of all birds were negative. No gastrointestinal parasites were found. Sarcocysts were described as follows:

Family Sarcocystidae Poche, 1913

**Fig. 1:**
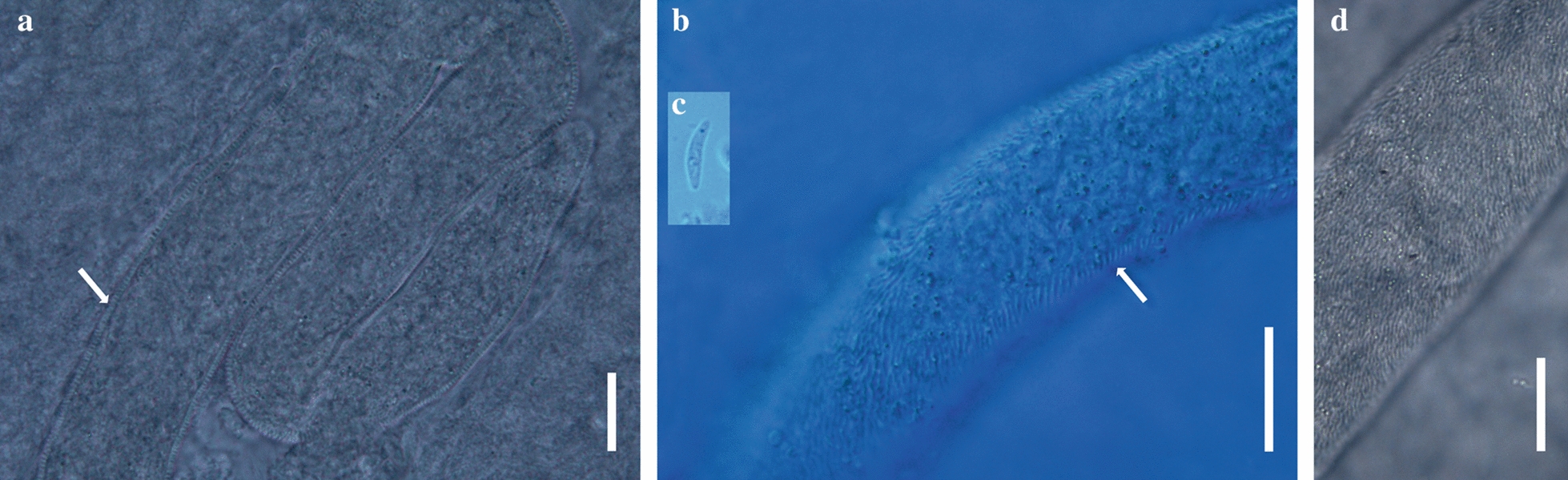
*Sarcocystis cristata* sp. nov. from *Corythaeola cristata*, light micrographs (**a**, **d**) and Nomarski interference-contrast photomicrographs (**b**, **c**) of wet mount: **a** Sarcocyst in breast muscle of host; **b** fragment of released sarcocyst (arrows indicate sarcocyst wall with finger-shaped villar protrusions); **c** bradyzoite; **d** villar protrusions on middle part of sarcocyst. Scale bars = 20 μm

**Fig. 2:**
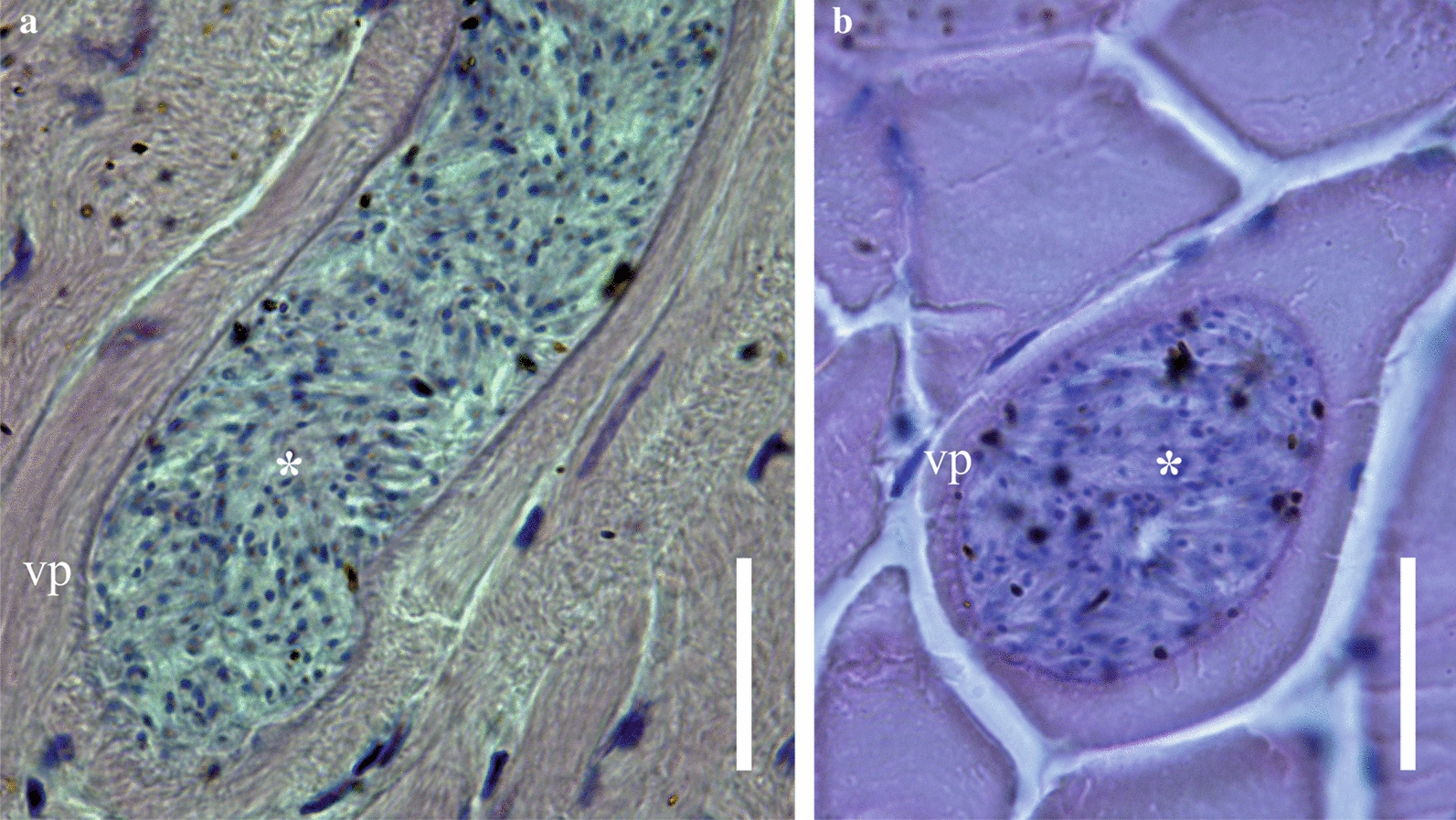
Haematoxylin and eosin-stained histological sections of skeletal muscle from *Corythaeola cristata*: Longitudinal (**a**) and transversal (**b**) sections of sarcocyst, respectively; asterisks indicate sarcocyst packed with bradyzoites. *vp* villar protrusions. Scale bars = 20 μm

*Sarcocystis cristata* sp. nov. (Figs. 1, 2)

Description: Microscopic, elongate, ribbon-shaped sarcocysts (Fig. 1a). The longest sarcocyst is 1348 long and 40 wide. Anterior and posterior ends rounded. The sarcocyst wall was characterised by the presence of finger-shaped villar protrusions, 2.6 long (Fig. 1b, d). Mature sarcocyst packed with some metrocytes and plenty of elongate, banana-shaped bradyzoites, 11.87–14.84 × 2.05–2.92 in size (*n* = 30) (Figs. 1c, 2a). There are some pyknotic nuclei around the sarcocyst, but without an apparent inflammatory reaction (Fig. 2a, b).

### Taxonomic summary

Intermediate host: Great blue turaco *Corythaeola cristata* (Vieillot, 1816) (Musophagidae).

Definitive host: Unknown.

Original distribution: Central Africa Republic.

Deposited material: Symbiotype (frozen muscle with sarcocysts) and genomic DNA in Eppendorf tube were stored at SVI Prague. GenBank sequences MT676454 (*18S* rRNA gene), MT676455 (*28S* rRNA gene), MT676453 (ITS1 region), MT681118 (*cox1* gene).

ZooBank registration: To comply with the regulations set out in article 8.5 of the amended 2012 version of the International Code of Zoological Nomenclature [18], details of the new species have been submitted to ZooBank. The Life Science Identifier (LSID) for *Sarcocystis cristata* sp. nov is urn: lsid:zoobank.org:pub:38C58A60-08C1-4CAD-B02F-A0C9B812E2B4

Etymology: The specific epithet is derived from the species name of its intermediate host, i.e. *cristata*.

Molecular sequences of the *18S* rRNA, *28S* rRNA, ITS1 and *cox1* loci from six sarcocyst isolates were successfully obtained with no intraspecific variability. Those sequences of *18S* rRNA were identical; therefore, only one of 1611 bp was submitted to GenBank (MT676454). Blasting showed high similarity between the *18S* rRNA sequence of *S*. *cristata* sp. nov. and those of *S*. *anasi*, *S*. *albifronsi* and *S*. *wenzeli* (99.8% each) in the mallard duck (*Anas platyrhynchos*) (EU553477), the white-fronted goose (*Anser albifrons*), both from Lithuania (EU502868) and chicken (*Gallus gallus*) from China (MT756990), respectively; *Sarcocystis* sp. isolate from chicken (most likely *S*. *wenzeli*, see [19]) (99.7%) from Brazil (MN845627); *S*. *rileyi* (99.5%) in the common eider (*Somateria mollissima*) in Norway (KJ396583); *S*. *atraii* and *S*. *chloropusae* (99.2% each) in the common coot (*Fulica atra*) (KJ810606) and the common moorhen (*Gallinula chloropus*) (KJ810604), respectively, both from Egypt. The *28S* rRNA locus (1350 bp) was most similar to those of *S*. *wenzeli* (99−99.2%) (MT756986-8), *S*. *albifronsi* (98.8%) (EF079885), *S*. *anasi* (98.7%) (EF079887), *S*. *chloropusae* (97.9%) (KJ810605), *S*. *rileyi* (97.2%) (KJ396585) and *S*. *atraii* (96.6%) (KJ810607). While sequences of the ITS1 region (1090 bp) were most similar to *S*. *wenzeli* (92.5−92.8%) (MT756994-7), *Sarcocystis* sp. isolate from chicken (91.2%) (MN846302), *S*. *anasi* (89.5%) (JF520779), *S*. *albifronsi* (88.4%) (JF520780), *S*. *chloropusae* (80.5%) (KJ810610), *S*. *rileyi* (78.2%) (KJ396584) and *S*. *atraii* (69.2%) (KJ810611). The *cox1* sequence (1013 bp) showed higher similarity to *S*. *albifronsi* (99%) (MH138310), *S*. *anasi* (98.7%) (MH138311), *S*. *wenzeli* (97.9%) (MT761700), *S*. *rileyi* (97.2%) (KJ396582) and *Sarcocystis* sp. isolate from chicken (97.1%) (MN848337).

**Fig. 3:**
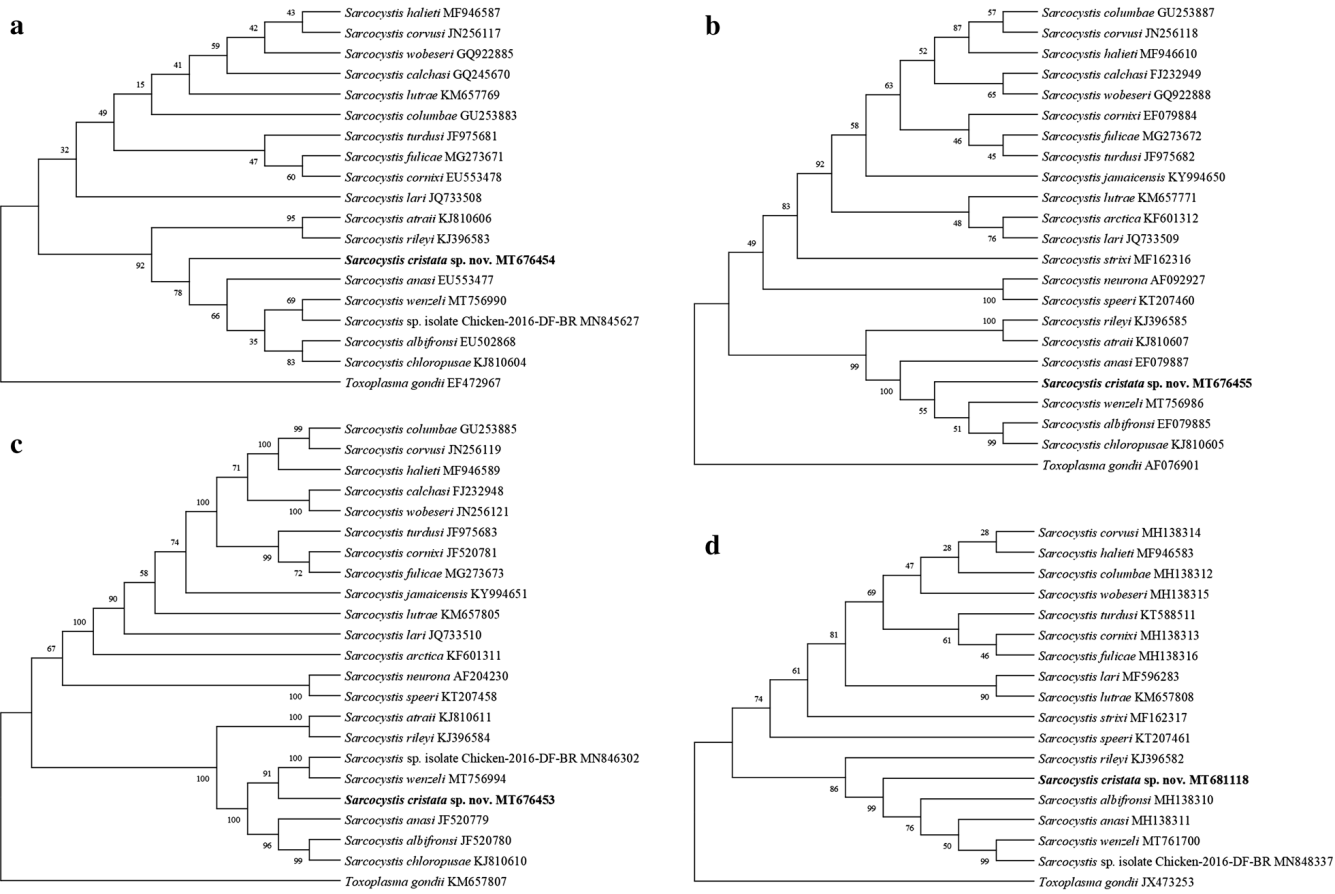
Phylogenetic trees of the species of *Sarcocystis* from some avian and mammal hosts based on **a** *18S* rRNA, **b** *28S* rRNA, **c** ITS1 and **d** *cox1* loci sequences. The numbers on phylogenetic trees represent bootstrap values based on 1000 replications. GenBank accession numbers follow *Sarcocystis* species

Phylogenetic analysis showed the relationships of the new species with other *Sarcocystis* spp. from birds as intermediate hosts. All four phylogenetic trees showed similar topologies regarding the formation of two groups, one formed mostly by *S*. *calchasi*, *S*. *columbae*, *S*. *cornixi*, *S*. *corvusi*, *S*. *fulicae*, *S*. *halieti*, *S*. *lari*, *S*. *lutrae*, *S*. *turdusi* and *S*. *wobeseri* and the second group with the new species and seven congeneric species (*S*. *albifronsi*, *S.* *anasi*, *S*. *atraii*, *S*. *chloropusae*, *S*. *rileyi*, *S*. *wenzeli*, *Sarcocystis* sp. chicken isolate). The position of *S*. *cristata* shows a stronger relationship with *S*. *wenzeli* and *Sarcocystis* sp. isolate chicken-2016-DF-BR (Fig. 3c), although it is also closely related to *S*. *albifronsi*, *S*. *anasi*, *S*. *atraii*, *S*. *chloropusae* and *S*. *rileyi* (Fig. 3a–d). *Sarcocystis atraii*/*S*. *rileyi* and *S*. *albifronsi*/*S*. *chloropusae* are sister species (Fig. 3a, c).

## Discussion

The thread-like shape of the present specimens and the asexual development in the striated muscles of the intermediate host, a heteroxenous life cycle and the presence of metrocytes and bradyzoites in the sarcocyst indicate that they belong to the genus *Sarcocystis* [20]. This parasite, despite the small sample size (*n *= 4), showed a relatively high prevalence in the examined birds, thus suggesting that its presence in turacos is possible and that the latter act as its natural intermediate host. This finding represents the first worlwide report of a *Sarcocystis* sp. in a member of the family Musophagidae.

As mentioned above, there are 26 valid species of *Sarcocystis* using birds as intermediate hosts, namely: *S*. *accipitris* Černá et Kvašňovská, 1986, *S*. *albifronsi* Kutkiené, Prakas, Sruoga et Butkauskas, 2012, *S*. *alectoributeonis* Pak, Sklyarova et Pak, 1989b, *S*. *alectorivulpes* Pak, Sklyarova et Pak, 1989b, *S*. *anasi* Kutkiené, Prakas, Sruoga et Butkauskas, 2012, *S*. *atraii* El-Morsey, El-Seify, Desouky, Abdel-Aziz, El-Dakhly, Kasem, Abdo, Haridy, Skai et Yanai, 2015, *S. calchasi* Olias, Gruber, Hafez, Heydorn, Mehlhorn et Lierz, 2010, *S. chloropusae* El-Morsey, El-Seify, Desouky, Abdel-Aziz, Sakai et Yanai, 2015, *S. columbae* Olias, Olias, Lierz, Mehlhorn et Gruber, 2010, *S. cornixi* Kutkiené, Prakas, Sruoga et Butkauskas, 2009, *S. corvusi* Prakas, Kutkiené, Butkauskas, Sruoga et Žalakevičius, 2013, *S. falcatula* Stiles, 1893, *S. fulicae* Prakas, Butkauskas, Švažas, Juozaityté-Ngugu et Stanevičius, 2018, *S. halieti* Gjerde, Vikøren et Hamnes, 2018, *S. horvathi* Rátz, 1908, *S. kirmsei* Garnham, Duggan et Sinden, 1979, *S*. *kutkienae* Prakas, Butkauskas et Juozaityté-Ngugu, 2020, *S. lari* Prakas, Kutkiené, Butkaukas, Sruoga et Žalakevičius, 2014, *S. lindsayi* Dubey, Rosenthal et Speer, 2001, *S. phoeniconaii* Göbel, Erber et Grimm, 1996, *S. ramphastosi* Dubey, Lane et van Wilpe, 2004, *S. rileyi* (Stiles, 1893) Minchin, 1903, *S. sulfuratusi* Dubey, Lane et van Wilpe, 2004, *S. turdusi* Kutkiené, Prakas, Butkauskas et Sruoga, 2012, *S. wenzeli* (Wenzel, Erber, Boch et Schellner, 1982) Odening, 1997, and *S. wobeseri* Kutkiené, Prakas, Sruoga et Butkauskas, 2010. Most of these species were morphologically described and apparently differ from the new species in the size of the sarcocyst and wall ultrastructure, although the first might be variable according to its age and the second was not analyzed in this study with transmission electron microscopy.

Molecular analysis of the four loci supported the identification of the present sequences as belonging to a new species. Groups of related species in the phylogenetic trees were formed, in one, mostly by *Sarcocystis* spp. with a bird of prey (e.g. hawks, eagles) as definitive host [see e.g. 9, 21–25], while the second grouped the new species and others allegedly using mammals as definitive hosts, such as *S*. *albifronsi* in Arctic fox (*Alopex lagopus*) from Lithuania [26], *S*. *anasi* and *S*. *rileyi* in *V*. *vulpes* and racoon dog (*Nyctereutes procyonoides*) from Lithuania [27] and Germany [28], *S*. *atraii* probably in American mink (*Neovison vison*) and red fox (*Vulpes vulpes*) from Egypt [29], *S*. *chloropusae* maybe in *V*. *vulpes* [30] and *S*. *wenzeli* experimentally in dogs and cats [19]. With the most conclusive locus (ITS1 region), the clade formed by *S*. *cristata* sp. nov. with *S*. *wenzeli* and *Sarcocystis* sp. isolated from chicken is well supported and shows the close relationship of these species. *Sarcocystis cristata* is a separate species from *Sarcocystis* sp. isolated from chicken and *S*. *wenzeli*, which are the same species, as stated by Pan et al. [19]. The latter two species and five other congeners formed a recurrent group of species reported in several previous works [e.g. 24, 29], despite being of different sarcocyst wall type (*S*. *albifronsi*, *S*. *anasi* and *S*. *wenzeli* type 9, *S*. *atraii* type 24, *S*. *chloropusae* type 10, *S*. *rileyi* type 23) and bird orders (e.g. Anseriformes, Galliformes, Gruiformes, Musophagiformes). Differences in the number and type of *Sarcocystis* species grouped with *S*. *cristata* sp. nov. are related to the availability of sequences in GenBank.

As mentioned above, the great blue turaco is a bird species endemic to West and Central Africa, where it apparently has few natural enemies. Therefore, the life cycle of *S*. *cristata* sp. nov. is unknown, but great blue turacos probably get infected after ingestion of sporocysts in food (leaves, buds, insects, flowers) or water, thus acting as intermediate hosts. Ants, cockroaches and crickets are food sources for many birds and can serve as paratenic hosts for internal parasites such as *Sarcocystis* [see 31]. The fact that the new species clustered with other avian *Sarcocystis* spp., whose definitive hosts are mammals, could indicate that some terrestrial carnivorous mammals (e.g. mongoose, linsang or side-striped jackal) might act as definitive hosts for it. These predatory mammals commonly occur in the same region and feed on arboreal birds, although nothing is known about their role as predators of turacos.

Shape, sarcocyst wall type and other morphological and morphometric data have been reported in almost all avian species, but they are insufficient to complete their specific identification [24]. In fact, Levine [20] wrote that “…the fine structure of the sarcocyst wall may change with age and is not considered necessarily satisfactory for separating species”. Recently, Prakas et al. [32] identified one morphological type of *Sarcocystis* in the herring gull *Larus argentatus* from Lithuania, but indeed it represented four different species. On the other hand, genetic markers have proved to be more reliable, although *18S* rRNA, *28S* rRNA and *cox1* were sometimes unable to separate species [see 9, 33]. For *Sarcocystis* species using birds as intermediate hosts, the ITS1 region was the most conclusive locus to clearly separate the new species from its congeners, while other loci were scarcely variable to distinguish species, although all genetic markers supported the designation of the new species. Apparently, the ITS1 region is more sensitive to the genetic differences among species, as mentioned by Prakas et al. [24] and Pan et al. [19], who also found a similar utility of molecular analysis while studying *S*. *fulicae* in *Fulica atra* from Lithuania and *S*. *wenzeli* in *G*. *gallus* from China, respectively.

The birds were probably infected before importation to Czech Republic, since they were in quarantine and then died. However, the presence of great turacos in zoos and private breeding facilities and their potential escape or release to the wild should be monitored, since these birds could spread sarcocysts to native carnivorous mammals in the region. The cause of death of turacos was neither determined, but the occurrence of *Sarcocystis* in birds should be diagnosed since related species, such as *S*. *rileyi* and *Sarcocystis* sp. isolated from chicken, have been reported as causing sarcocystosis in the skeletal muscle (rice breast) of ducks [34] and meningoencephalitis in the brain of chickens [35], respectively. Future studies may be focused on the proper parasitic diagnosis before importation, thus preventing the economic losses related to the trade of turacos around the world.

## Conclusions

To our best knowledge, this is the first study focused on the sarcocysts of a musophagiform bird worldwide. Genetically, *S. cristata* sp. nov. from the great blue turaco represents a distinct species. Phylogenetic analyses are useful for predicting potential definitive hosts of the new species, although future studies should be focused on faecal or intestinal samples of possible native definitive hosts (mammals) to elucidate their role in the life cycles of *Sarcocystis* species.

## Data Availability

The sequences generated in the present study were submitted to the GenBank database under the accession numbers MT676453–MT676455, MT681118.

## Abbreviations

bp: Base pairs
cox1: Cytochrome *c* oxidase subunit 1
DNA: Deoxyribonucleic acid
ITS1: Internal transcribed spacer 1
NCBI: National Centre for Biotechnology Information
PCR: Polymerase chain reaction
rRNA: Ribosomal ribonucleic acid
sp. nov: New species
